# Predicting the effect of individual weight-bearing on tibial load and fracture healing after tibial plateau fractures–introduction of a biomechanical simulation model

**DOI:** 10.3389/fbioe.2025.1659029

**Published:** 2025-09-25

**Authors:** Annchristin Andres, Michael Roland, Kerstin Wickert, Stefan Diebels, Daniel Truhn, Tina Histing, Benedikt Braun

**Affiliations:** ^1^ Applied Mechanics, Saarland University, Saarbrücken, Germany; ^2^ Department of Diagnostic and Interventional Radiology, University Hospital Aachen, Aachen, Germany; ^3^ Faculty of Medicine, BG Hospital Tuebingen, University Hospital Tuebingen on Behalf of the Eberhard-Karls-University Tuebingen, Tuebingen, Germany

**Keywords:** partial weight bearing, interfragmentary movement, construct stability, musculoskeletal simulation, proximal tibia fracture, motion capturing

## Abstract

**Purpose:**

The prescribed amount of weight-bearing after tibial plateau fractures is controversial because it affects osteosynthetic construct stability and fracture healing. We aim to introduce a simulation model that adequately predicts the effects of different weight-bearing amounts on stability and healing, based on the patient’s individual fracture pattern and treatment construct.

**Methods:**

To safely test different amounts of weight-bearing limits, we first extracted knee joint forces for different weight-bearing limits from musculoskeletal simulation based on monitoring data of 22 uninjured participants. Correct loading was ensured with a force-measuring insole. We then tested three patients after tibial plateau fracture with their current weight-bearing level and constructed a simulation model determining implant stress, knee joint force, and fracture gap interfragmentary strain. The patient-specific weight-bearing level was then substituted for weight-normalized uninjured participant data to test different weight-bearing levels in the simulation model.

**Results:**

The simulation model calculated individual construct stiffness and interfragmentary strain at different weight-bearing levels following the clinical course. When comparing the patient’s individual weight-bearing input with the weight-normalized input of the uninjured participants at the same level, comparable knee joint forces were extracted, showing the feasibility of this approach.

**Conclusion:**

Using an adapted reference movement database, the model allows the determination of safe weight-bearing ranges concerning construct stability and fracture healing based on individual fracture morphology and treatment without exposing patients to excessive weight-bearing. Future studies can test this approach in more extensive patient-number studies and different treatment situations.

## 1 Introduction

Tibial plateau fractures represent about 1% of all fractures in adults and are considered among the most complex joint injuries to be treated (Court-Brown and Caesar, 2006; Delp et al., 2007). Resulting from high-energy mechanisms in younger patients, as well as low-energy trauma with associated poor bone quality in the elderly, this fracture entity is associated with a limited prognosis due to cartilage, as well as additional soft tissue injury (Arnold et al., 2017; Gaston et al., 2005; Neal et al., 2017). While the specific osteosynthetic construct, depending on the fracture configuration, is still debated, the mainstay of treatment is open reduction and internal fixation (Canton et al., 2022; Mcnamara et al., 2015). According to the AO recommendations for weight-bearing after tibial plateau fracture treatment, patients should be kept non-weight-bearing for 10–12 weeks. However, a recent survey analysis among Dutch trauma surgeons has shown that this recommendation is only inconsistently followed by surgeons and that the prescribed start of at least some weight-bearing mostly ranges between 0 and 6 weeks (van der Vusse et al., 2017). Furthermore, the general compliance of patients to non- or limited weight-bearing regimens is limited in several studies (Braun et al., 2017; Chiodo et al., 2016; Kammerlan et al., 2018). In addition, current studies have suggested that some degree of early or even permissive full weight bearing may be safe in select cases (Canton et al., 2022; The et al., 2017; Williamson et al., 2018).

Multiple studies have focused on determining a safe range for weight-bearing after these injuries, showing that at least a moderate amount of weight-bearing early on is not secure with standard open reduction internal fixation constructs, but might be beneficial for the patient regarding reported outcomes (Arnold et al., 2017; Canton et al., 2022; Iliopoulos and Galanis, 2020; Pishtiwan Hassan Shaker Kalmet et al., 2019; Williamson et al., 2018). Studies have gone so far as to recommend permissive weight-bearing, according to the patient’s comfort, but even then, patients reached full weight-bearing on average only after 14 weeks (Pishtiwan Hassan Shaker Kalmet et al., 2019). Recent randomized and comparative studies demonstrated that immediate or earlier weight bearing after internal fixation can lead to superior functional outcomes without increased radiological complications, and that earlier loading does not increase complication rates or compromise fracture union, further questioning the necessity of prolonged restriction (Erick et al., 2023; Ibrahim et al., 2025). Depending on the fracture type and treatment, there is limited evidence that higher average joint reaction forces at the early point can lead to an increase in intra-articular step-off formation, thus showing the clinical need for a reliable tool to determine safe ranges of weight-bearing on an individual level (Thewlis et al., 2015).

To accurately determine the safe range of weight-bearing after tibial plateau fractures, musculoskeletal simulation can help assess individual construct stability. This is a reliable method to assess implant stresses, joint reaction forces, and interfragmentary fracture strain in other lower extremity fracture entities (Braun et al., 2021; Marmor et al., 2020). While standard models of the proximal tibia have already been constructed to test different mechanical conditions in fracture models (Carrera et al., 2018; Carrera et al., 2016), to our knowledge, no model has been developed to calculate individual, case-specific construct stability and the resulting interfragmentary strain. Previous research often relies on generic or population-averaged models that do not incorporate patient-specific anatomical data or individualized loading conditions (Cunningham et al., 2017). These standard models typically apply simplified loading assumptions, uniform implant configurations, or generalized boundary conditions that may not accurately reflect the biomechanical environment of a healing fracture in a specific patient.

The aim of the current study was thus to construct a simulation model that adequately predicts the effect of different weight-bearing amounts on the stability and interfragmentary strain of fracture treatment, based on the patient’s individual fracture pattern and treatment construct.

## 2 Materials and methods

This study recruited 22 healthy subjects, including 11 women and 11 men with no history of lower extremity fracture, musculoskeletal disease, or neurological impairment. These individuals served exclusively as a normative reference group for musculoskeletal simulation input data. In contrast, the clinical part of the study involved three postoperative patients with tibial plateau fractures treated by open reduction and internal fixation, who were included to test the feasibility of the patient-specific simulation workflow. We used the Xsens™ system, known for its precision in recording human movement, to capture motion data. The participants actively participated in the Timed Up and Go (TUG) test, a standardized assessment that measures their mobility and functional capacity (Podsiadlo and Richardson, 1991). During the TUG test, individuals had to rise from a seated position, walk a short distance, turn around, and return to the seated position, with the primary outcome being their completion time, measured in seconds. Before conducting the test, we provided all participants with a comprehensive explanation of the TUG procedure and its significance. We alternated the partial load conditions between subjects, with one group (n = 11) of 20 kg and the other group (n = 11) an equivalent load of half their body weight as partial weight bearing. To ensure participants adhered to their assigned partial weight, we actively employed a Moticon™ insole system to monitor and record the load during the TUG test. We actively instructed participants in properly handling crutches using the 3-point gait method to ensure consistency and safety.

Furthermore, participants actively assessed their perception of the partial load using a household scale. Before the formal testing, all participants walked over the scale ten times to familiarize themselves with the sensation of the ordered partial load. We conducted the TUG test in two rounds: first, with a full load to establish reference values, and then with the arranged partial load, determined by the subject’s group assignment (20 kg or half body weight). This dual-round approach allowed for a comprehensive evaluation of each participant’s performance under full and partial load conditions, enabling an active comparison of their mobility and functional capacity in different load scenarios. The TUG test, a quick, reliable, and standardized assessment, actively evaluated participants’ mobility and functional capacity throughout this study (Izzah et al., 2019; Kristensen et al., 2007).

**FIGURE 1:**
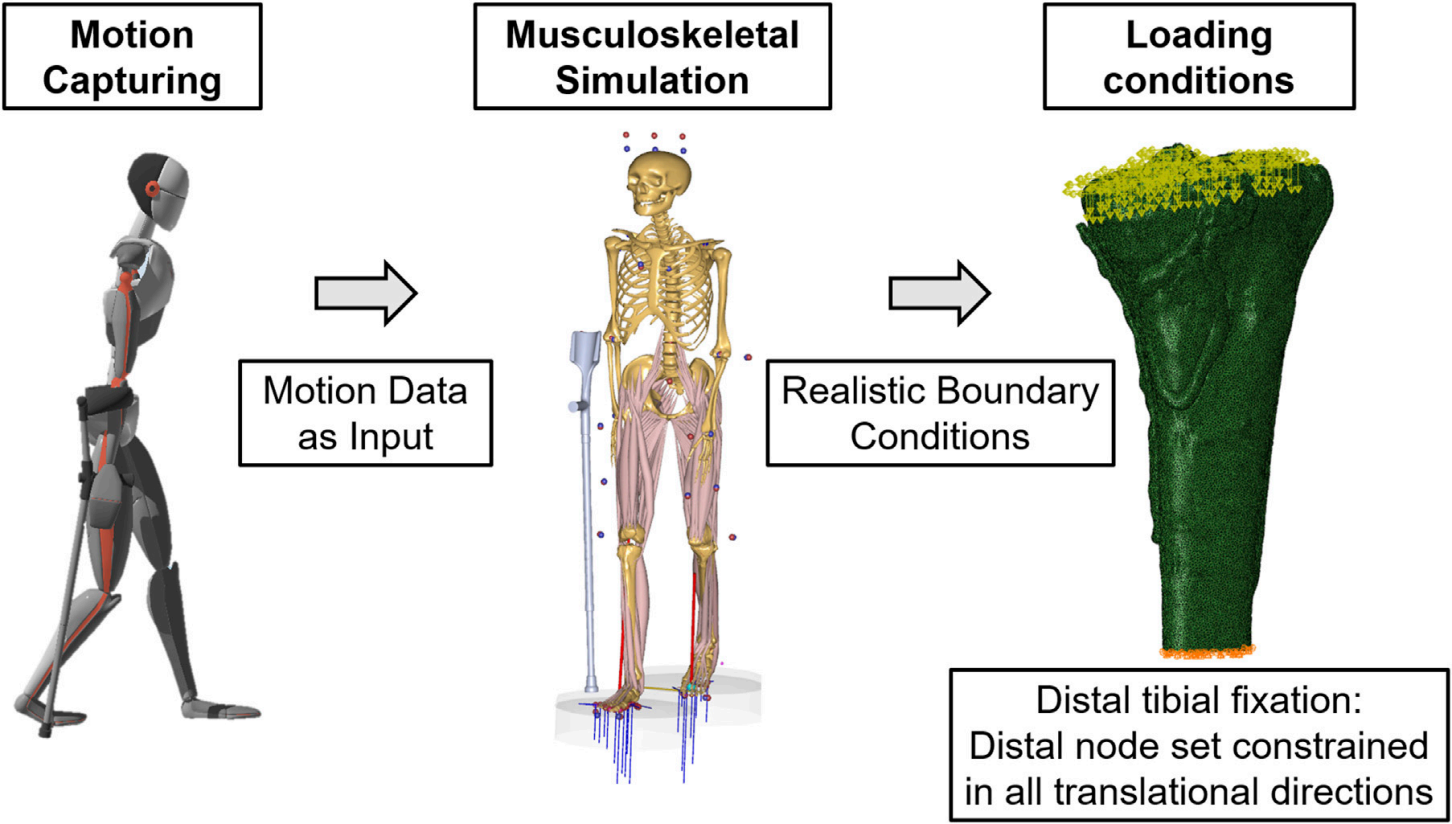
Integrating the Xsens™ motion capturing system with AnyBody™ uses input data from captured movements and the subsequent realistic boundary conditions to simulate knee joint reactions.

The workflow simultaneously measures the subject using the Xsens™ motion capturing system (Supplementary Figure S1), Moticon™ measurement insoles (Supplementary Figure S2), and Novel™ loadpads (Supplementary Figure S3). The Xsens™ motion data is exported as. bvh (BioVision Hierarchy) files and imported into AnyBody™ software (Supplementary Figure S4), a musculoskeletal simulation tool that works seamlessly with Xsens™. Data from the measurement insoles and loadpads are imported as. txt files into AnyBody™, where the musculoskeletal model is scaled to the subject’s anthropometric data. This data is then used to calculate the joint and muscle forces inversely. The interface between Xsens™ and AnyBody™ ensures seamless data integration. Xsens™ gait data is exported as. bvh files for each subject and processed in AnyBody™ using the internal AMMR (AnyBody™ Model Repository), an open collection of examples and models of the musculoskeletal system, to map the gait cycle accurately. After completing the motion data acquisition with the 22 healthy subjects using the Xsens™ system, the data were analyzed using AnyBody™. Based on the recorded data, AnyBody™ allows us to perform a detailed analysis of joints, muscles, and moments. This analysis is critical for evaluating the effects of partial loads, particularly on knee forces, as part of our study of tibial plateau fractures (Figure 1). The knee joint forces included for a controlled partial load (20 kg, or 50% of body weight) were derived from healthy individuals. Patient data were used to determine the force values for full weight-bearing. To allow for inter-subject comparison, all computed knee joint reaction forces from the AnyBody™ musculoskeletal model were normalized to each participant’s body weight (%BW). This was done by dividing the joint force values (in Newtons) by the individual’s body weight (in Newtons) and multiplying by 100. The resulting values are expressed as a percentage of body weight (%BW) and plotted across the gait cycle. The systems used for inertial motion capture (Xsens™), insole-based force measurement (Moticon™), and musculoskeletal modeling (AnyBody™) provide a validated and reproducible workflow, as demonstrated in previous studies (Burns et al., 2019; Andersen et al., 2009; Muro-de-la-Herran et al., 2014). Detailed instrumentation specifications are in the Supplementary Material.

**FIGURE 2:**
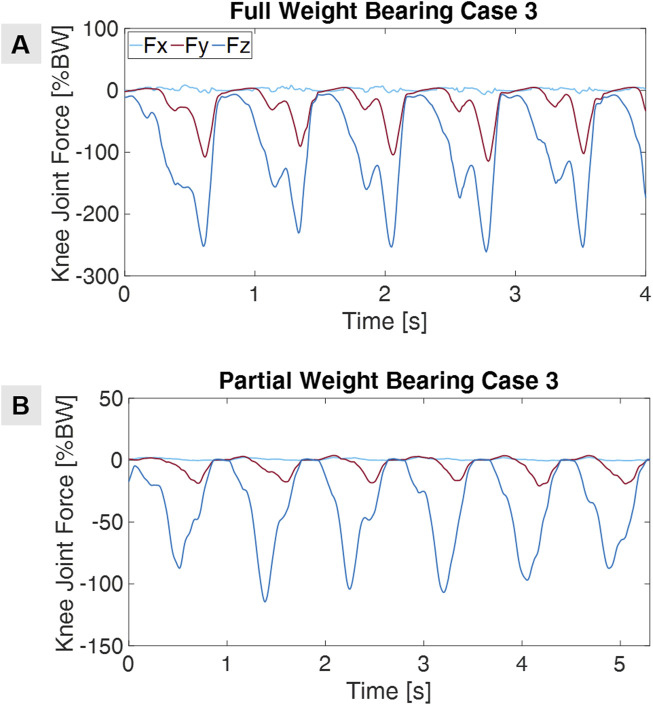
Knee joint forces for full **(A)** and partial **(B)** weight-bearing in use case 3.

Furthermore, our research goes beyond healthy subjects as we actively measure actual patients with their prescribed partial weight-bearing. We follow a similar measurement workflow to healthy subjects and perform these measurements in the clinic using the Xsens™ motion measurement system. As with healthy subjects, the data obtained from the patients is also analyzed using AnyBody™ software. Figure 2 illustrates an example of knee joint force curves for a patient, using his motion-capturing data. Additionally, we utilize the patients’ CT images to generate patient-specific 3D models through segmentation and downstream image processing, which are then used in finite element (FE) simulations. This approach enables us to create a database of partial loads, providing insight into how various loads impact the patient’s musculoskeletal dynamics, with a particular focus on tibial plateau fractures and their treatment.

The first step in the FE analysis methodology is to create geometric models using the individual DICOM image stack of the patient’s postoperative CT scan and a six-rod bone density calibration phantom (QRM-BDC/6, QRM GmbH, Moehrendorf, Germany) (Braun et al., 2021). Therefore, the images are segmented into masks using an adaptive thresholding procedure based on the calibration phantom. The calibration phantom with known density values establishes a relationship between Hounsfield Units and bone mineral density. After completing the segmentation, the treating trauma surgeon visually controlled the segmentation results of the fracture gap and made corrections if necessary. Then, we create high-resolution adaptive finite element meshes using the FE Mesher from Simpleware™ (Simpleware™, Synopsys, Mountain View, United States), employing C3D10 elements and defining node sets at the distal and proximal ends of the bone. Specify the homogeneous materials with standard properties within Simpleware™, sourcing values for the implant and fracture gap from the literature (Claes and Heigele, 1999; Imam and Fraker, 1996). To establish the relationship between elasticity and bone density, map the grayscale values of the CT data to the Hounsfield scale and mechanical local bone properties (Cattaneo et al., 2001; Hvid et al., 1989; Rho et al., 1995; Zannoni et al., 1999). The exact workflow is outlined in the Supplementary Material, including additional information about the mesh (Supplementary Figure S5). Following previous studies, assume an isotropic heterogeneous material with a varying Young’s modulus and a fixed Poisson ratio (Nir et al., 2009; Yosibash et al., 2007). Define cortical and trabecular bone mapping based on local ash density and equivalent mineral density, as described in prior research (Braun et al., 2021; Brent Edwards et al., 2013; Helgason et al., 2008; Knowles et al., 2016). Pass all material properties to the FE meshes, storing them in the nodes and elements of the corresponding masks. After setting up node sets, elements, and material properties in Simpleware™, create the mesh and export it as a. inp file.

Only descriptive statistics (means, standard deviations) were reported; no inferential testing was performed due to the small sample size.

### 2.1 Simulation workflow

Finite element analysis was conducted using Abaqus/CAE 2020, Abaqus™ (Dassault Systems, Velizy-Villacoublay, France). The input file generated in Simpleware™, which contained the finite element mesh, material properties, and predefined node sets (distal and proximal), was imported into the software. To simulate fixation, a displacement/rotation boundary condition was applied to the distal node set, fully constraining it in all three translational directions (U1, U2, and U3). Time-dependent knee joint forces derived from a musculoskeletal simulation in AnyBody™ were implemented in Abaqus using tabular amplitude definitions. Separate amplitude curves were created for each force component (Fx, Fy, and Fz). These forces were applied as concentrated loads to the proximal node set, each aligned with the corresponding spatial direction. Next, a static analysis step was defined with a time period matching that of the applied amplitudes. Nonlinear effects were disabled, and appropriate incrementation settings were selected to ensure stable convergence. For postprocessing, field output requests included the stress tensor (S), von Mises stress (MISES), strain tensor (E), displacement (U), reaction force (RF), and integration point volume (IVOL). Finally, a simulation job was created and submitted for execution. The simulation follows linear elastic assumptions.

For the simulation of Interfragmentary movement, we focus on a mechanobiological approach based on mechanical stimuli, as discussed in the works of Claes et al. (1998), Shefelbine et al. (2005). Specifically, we examine the interaction between octahedral shear strain, which is derived from the deviatoric component of the strain tensor related to shape distortion, and volumetric strain, which is associated with volume changes and hydrostatic pressure (Ghiasi et al., 2017). This approach emphasizes the significance of mechanical deviatoric strains in influencing cell differentiation and tissue formation. This notion aligns well with experimental findings (Ito et al., 2003; Doblaré et al., 2004; Richardson et al., 2003). To precisely analyze the mechanics within the fracture gap, we read the strain tensor for each tetrahedral element in that region. Both octahedral shear strain and volumetric strain were calculated and assessed according to the parameters established by Claes et al. (1998), Shefelbine et al. (2005). A MATLAB script is used to convert ODB (Output Database) files, produced by Abaqus, to store simulation results, into VTK (Visualization Toolkit) files, enabling improved visualization and analysis through advanced post-processing tools. Further information about the simulation process can be found in the Supplementary Material.

### 2.2 Case data

#### 2.2.1 Use case 1

The first case is a 57-year-old patient suffering from a closed proximal tibia fracture (AO/OTA 41 C1.2; Schatzker IV) after falling from a ladder. Initially treated at an external institution, the patient was introduced to our department with a posteromedial malreduction, resulting in an unreduced knee joint with posteromedial dislocation and a bone non-union. The patient underwent revision with an open reduction and fixation using a medial, posteromedial, and anterolateral locking plate, along with a medial tricortical allograft. Postoperatively, a 20 kg partial weight-bearing order was given. The patient experienced an uneventful healing course, regaining full weight-bearing capacity and demonstrating timely fracture consolidation, with high satisfaction.

#### 2.2.2 Use case 2

The second case is of a 72-year-old patient with atrial fibrillation, hypertension, and hypothyroidism, who sustained a closed proximal tibia fracture in a fall from standing height (AO 41 B3.3h; Schatzker IV). She was treated with an open reduction and medial locking plate osteosynthetic fixation. Postoperatively, a 20 kg partial weight-bearing order was given. Initially, the patient exhibited delayed healing, with early screw-back failure at the 6-week follow-up. Upon further review of the history, while the patient attempted to comply with the weight-bearing recommendation, she reported difficulties maintaining balance, which may have contributed to an overload of the fracture situation. At this point, she was instructed to limit weight-bearing for another 6 weeks with only gradual increases under strict physical therapy guidance. The fracture then showed slow but steady consolidation over the next 6 months. The screw progressively backed out and was removed 3 months after the initial treatment.

#### 2.2.3 Use case 3

Case three is a 52-year-old patient who sustained a closed proximal tibia fracture in a skiing accident (AO 41 C3.1e; Schatzker IV). She was treated with a medial, posteromedial, and anterolateral locking plate with an open reduction utilizing a lateral epicondylar osteotomy. Postoperatively, a 20 kg partial weight-bearing order was given. The patient exhibited an uneventful healing course, with timely fracture consolidation and a favorable clinical outcome. Accordingly, the simulation predicted an uneventful healing course during all weight-bearing levels.

For all three patients, the clinical prescription was an immediate postoperative partial weight-bearing of 20 kg. Higher load levels (50% body weight and full weight-bearing) were not prescribed clinically but were tested only virtually in the simulation model.

## 3 Results

This section first presents the results of the diagrams of the ground reaction forces during the TUG test, focusing on compliance with partial weight-bearing recommendations in healthy test subjects. The derived mean force curve of the knee joint, based on the analyzed AnyBody™ data, is then examined. This assessment facilitates a deeper understanding of the impact of partial weight-bearing on joint forces and, consequently, its influence on interfragmentary motion in patients. We then examine patient outcomes and the effects of different loading scenarios on design stiffness and interfragmentary motion. Here, the analysis focuses on the mechanical effects of different partial weight-bearing approaches and evaluates their effectiveness in promoting healing. This analysis contributes to a more nuanced understanding of the effectiveness of orthopedic treatments and bridges the gap between theoretical modeling and clinical application.

### 3.1 Healthy subjects

**FIGURE 3:**
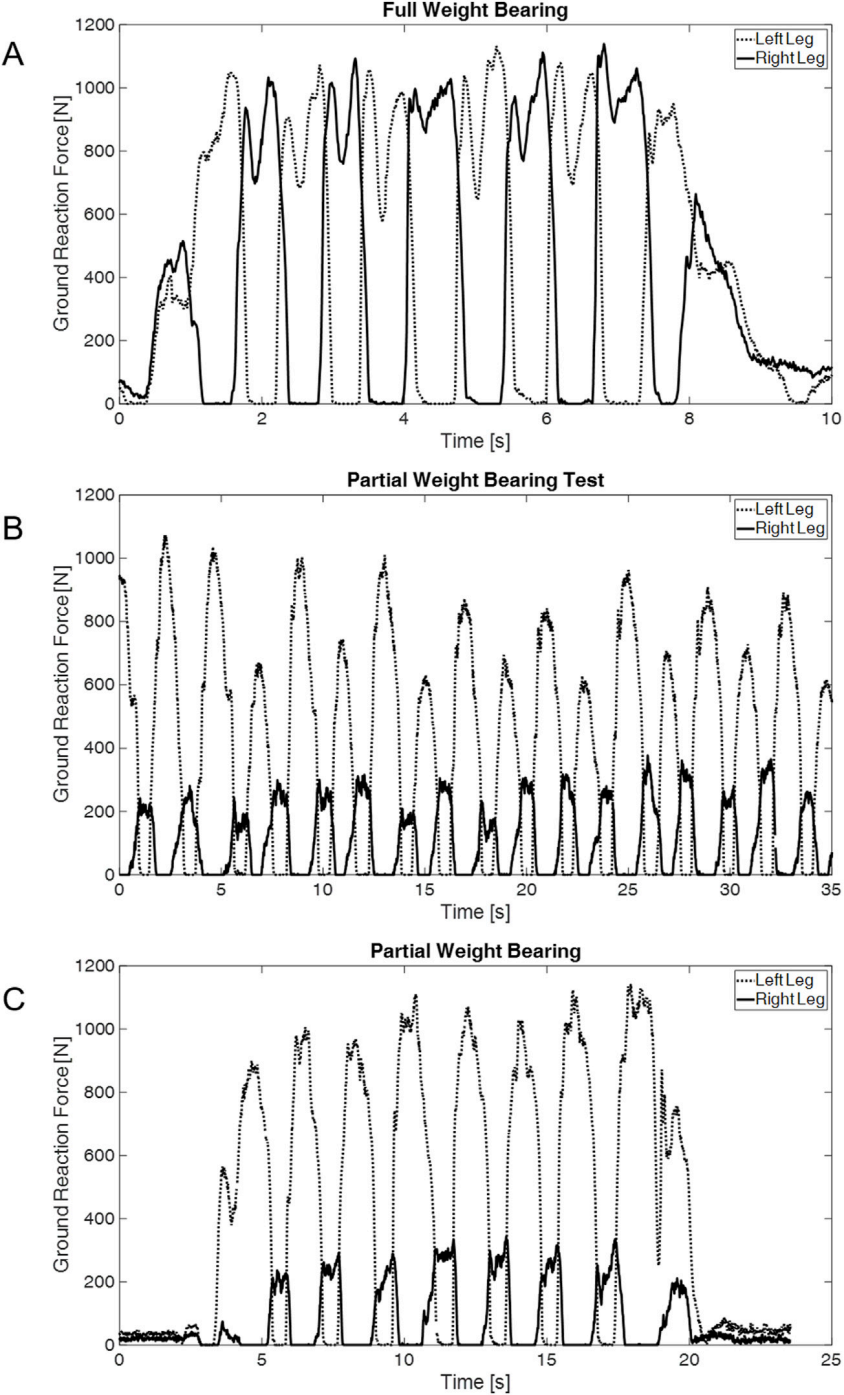
Ground reaction force curves for **(A)** full weight-bearing, **(B)** Training with partial weight-bearing and crutches, and **(C)** partial weight-bearing reprehensive for one subject.

**FIGURE 4:**
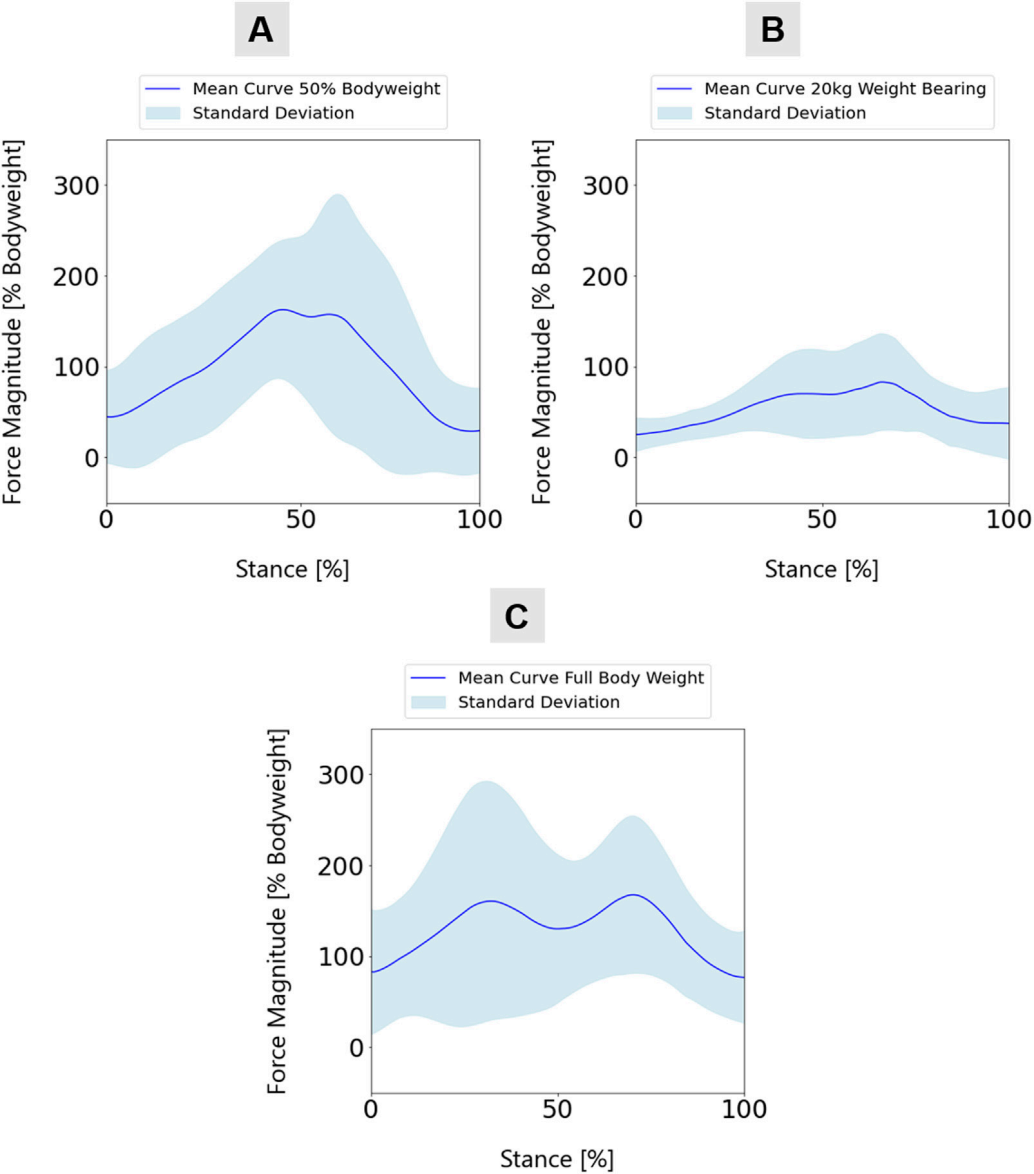
Mean knee joint force curve over all participants (n = 22) for **(A)** 20 kg weight-bearing, **(B)** 50% body weight, and **(C)** full weight-bearing and natural walking.

A total of 22 participants underwent testing, comprising 11 women and 11 men. The average age of the participants was 26 ± 4 years. The mean weight is 71 ± 15 kg, and the average height is 176.5 ± 11 cm. The testing protocol involved each participant undergoing 11 walking trials with 50% of their body weight, followed by ten repetitions with a partial load of 20 kg. Figure 3 illustrates a representative instance of the ground reaction forces of the left and right feet obtained from insole measurements during sessions with healthy test subjects. The results of the mean knee joint forces, which were standardized to the body weight of the test subjects for comparison purposes, are illustrated in detail in Figure 4. It shows the mean knee joint force as a percentage of body weight during a gait cycle, initiated and completed by heel strike, with a partial load of 20 kg or 50% of the subjects’ body weight. The testing commenced with recording the TUG task during normal walking (Figure 4A). Subsequently, the subject walked with crutches and underwent testing for partial weight-bearing using a household scale. The subject underwent repetitions of partial weight-bearing trials of 20 kg on the right foot, consistent with standard clinical protocols (Figure 4B). Following these trials, the TUG task was repeated under the prescribed partial weight-bearing conditions, evaluating the ground reaction forces associated with this training regimen (Figure 4C). A more detailed representation of the knee joint forces can be seen in Supplementary Figure S6. Therefore, partial weight-bearing was implemented in the AnyBody™ system, and inverse dynamics were performed to infer the respective joint forces. The implementation utilized the measured ground reaction force from Moticon™ in the gait model, along with the corresponding gait data from the Xsens™ measurement. This detailed analysis reveals the nuanced impact of partial load on knee joint forces. However, the differences between the test subjects, particularly at 50% of body weight, demonstrate the individual nature of the effects of partial loading on knee joint forces. An example is the stark contrast between the impact of 50 percent partial weight-bearing on subjects with a body weight of 50 kg and those with a body weight of 90 kg. Furthermore, it is crucial to recognize that a partial load of 20 kg can increase knee joint forces to a peak of 100% of a person’s body weight, highlighting the significant impact that even relatively small external loads can have on knee joint biomechanical loads.

### 3.2 Patient-specific simulations

**FIGURE 5:**
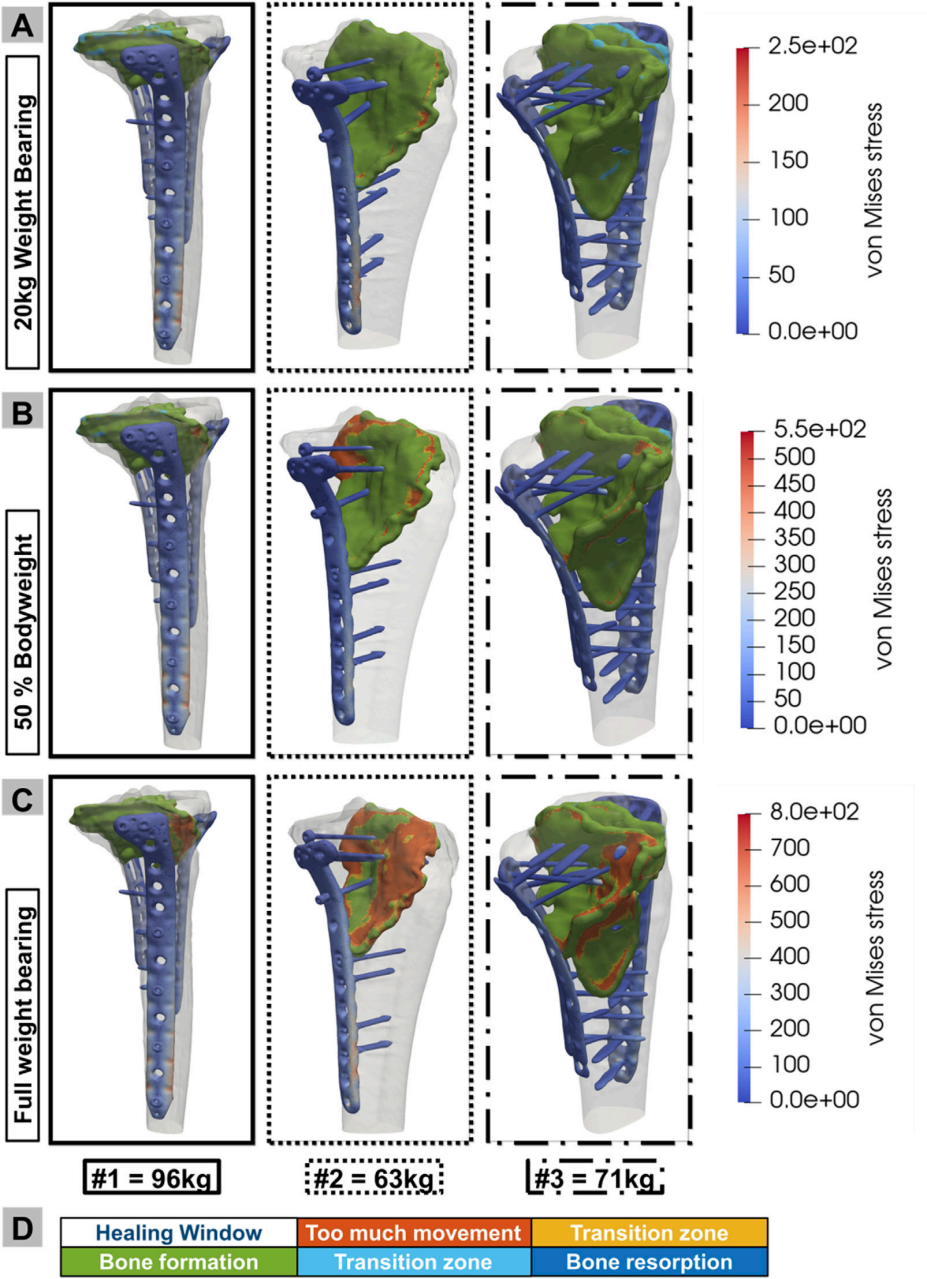
Results of the biomechanical simulation of digital twins for use cases 1, 2, and 3 under three different loading conditions: **(A)** 20 kg partial weight-bearing, **(B)** 50% body weight, and **(C)** full weight-bearing. Additionally, the implant stress in MPa is included for the different loading conditions. **(D)** Color pattern of the healing window, illustrating the quantity and precise position of the elements in the various healing states.

To investigate the effects of partial weight-bearing on different fracture scenarios and treatment modalities, digital twins representing the three patients were subjected to finite element simulation. These simulations replicate the biomechanical effects of various loading conditions on the patient’s lower extremities, simulating a gait cycle with partial loading of 20 kg, 50% of the patient’s body weight, and a full weight-bearing scenario. Clinically, all patients were limited to 20 kg partial weight-bearing postoperatively; the 50% body weight and full weight-bearing conditions were included solely as virtual test scenarios in the simulation. The methodology for applying these loads graphically, as shown in Figure 1, provides a clear visual reference for the biomechanical conditions applied. With the boundary conditions of the actual patient measurement, comparable results are obtained; however, these are limited by the variable testing of the partial load. Figure 5 presents the outcome of these simulations, with the data for the three patients arranged horizontally from use cases 1 to 3. In particular, the analysis considers the stresses and strains in the fracture gap. It interprets these variables through the bone healing window (Braun et al., 2021; Lutz, 2021; Lutz, 2017; Shefelbine et al., 2005) by linking local stresses and strains in the fracture gap, as described by Claes and Heigele (1999) (Figure 5D) - a concept that relates the mechanical environment of a fracture site to the potential for bone healing. Reducing interfragmentary motion at full weight-bearing improves fracture stability. In addition, the simulation output von Mises stress analyzed stresses within the plate construction. In all three patient cases, the maximum von Mises stresses were below the material-specific yield strength of medical titanium aluminum alloys (Geetha et al., 2009), indicating sufficient construct stability.

• Case #1: The simulation results predicted an uneventful healing course after the revision of all weight-bearing levels. The maximum value is below 400 MPa with uniform stress distribution, indicating increased stability when supplied with three plates.
• Case #2: High local shear stresses outside the defined healing window under full weight-bearing. The full load caused locally increased stresses (>600 MPa) in the area of the distal screws of the medial plate.
• Case #3: The simulation predicted an uneventful healing course at all weight-bearing levels. The stress values in all plates were consistently below 500 MPa.

### 3.3 Clinical outcomes

Clinical outcomes aligned with the simulation predictions:

• Case #1: Healed uneventfully, consistent with patient-specific simulation outcomes.
• Case #2: Clinically observed retraction of a screw indicates a mechanically critical situation, which clinicians addressed by adjusting the post-treatment. The course went on to fracture consolidation only after adaptation to her weight-bearing and physical therapy training.
• Case #3: Healing occurred without major complications. The results of the limit simulation under higher loads corresponded to the cautious clinical progression of the load required for this case.

## 4 Discussion

The presented results demonstrate a simulation approach for determining a safe range of postoperative weight-bearing in individual patients after tibial plateau fractures, considering construct stability and interfragmentary strain that influence fracture healing. Notably, this model integrates the patient’s individual fracture and treatment situation with weight-adjusted movement norm data from the uninjured reference group, enabling the safe testing of different weight-bearing scenarios without exposing the patient to potentially harmful situations. Furthermore, construct stability is ensured for every patient by analyzing the von Mises stress, which remains below the critical material limits (i.e., yield strength of ∼880 MPa) for titanium aluminum alloys used in medical-grade implants (Geetha et al., 2009). This guarantees that no threshold values are exceeded and the components are not subjected to excessive stress. In addition, the analysis shows that by increasing the number of implants, patients 1 and 3 had a mechanical stimulus in the lower part of the ranges expected for uneventful bone healing, indicating a favorable biomechanical environment for fracture healing. This improvement in stability is particularly beneficial for bone healing, as it reduces the mechanical stimulus at the fracture site and brings it into the optimal range of the healing window.

The feasibility and validity of the presented simulation approach are grounded in a series of previous methodological and clinical studies that have established reliable workflows for biomechanical fracture simulation. Braun et al. (2021) demonstrated the integration of motion capture in tibial pseudarthrosis treatment, introducing a patient-specific approach to assess fracture mechanics. Similarly, the automated CT-based finite element pipeline by Dahmen et al. (2015) enabled individualized simulation of bone-implant constructs and laid the groundwork for reproducible, image-based simulation methods. These technical principles were further refined by Roland et al. (2024), who proposed a direct algorithmic strategy for simulating bone healing based on CT imaging, and by Orth et al. (2023), who used simulations to assess the effects of motion and weight-bearing on fracture healing in the lower leg. The applicability of the measurement systems used in cases of tibial fractures was 21 demonstrated by Andres et al. (2025a) through weekly measurements during the healing phase. The current study extends these approaches by incorporating individualized musculoskeletal loading, derived from inertial motion capture and force-sensing insoles, and fracture-specific finite element modeling to create digital twins of postoperative patients. This enables the realistic simulation of weight-bearing scenarios without exposing patients to biomechanical risk, aligning with earlier feasibility studies that utilized dynamic *in vivo* load measurements and simulation-based analysis of fracture healing (Andres et al., 2025b; Braun et al., 2022). Concerning validation, the predictive power of the simulation model is supported by agreement with clinical outcomes in all three use cases, and notably, in case #2, simulation results under full weight-bearing conditions predicted localized overloading and interfragmentary shear strain outside the safe healing window, which corresponded to screw loosening and delayed consolidation observed in the clinical course. We acknowledge that delayed consolidation in this patient was multifactorial, influenced by age, comorbidities, and surgical construct, in addition to biomechanical factors. Nonetheless, the simulation highlighted overload at the fracture site consistent with the clinical finding of screw loosening, indicating that while the model cannot exclude non-mechanical contributors, it reliably identified a key mechanical risk factor. These findings are consistent with the mechanobiological principles of Claes and Heigele (1999) and Shefelbine et al. (2005), which establish threshold values for strain-driven tissue differentiation during bone healing. Moreover, recent experimental validation of interfragmentary motion in bone-implant systems, such as by Wickert et al. (2024), reinforces the technical accuracy of the FEM-based predictions. In addition, the use of validated software environments (e.g., AnyBody™ and Abaqus™) and standardized imaging-to-simulation pipelines (Augat et al., 2021) further enhances the reproducibility and transferability of the approach. The current model builds upon and extends a robust methodological foundation, demonstrating technical feasibility and clinical validity in predicting mechanical outcomes relevant to postoperative fracture care.

The recording of joint forces in healthy test subjects enables a differentiated understanding of the biomechanical effects of partial loading during walking. Detailed measurements of knee joint forces can be found in the OrthoLoad™ database (Heinlein et al., 2009). Nine patients were measured using an instrumented knee joint implant while performing various activities. However, the database only provides two data sets, k5r and k2l, during a 3-point walk. This innovative approach circumvents the limitations associated with the need for measurable implants, giving valuable insights into the effects of loading during various walking scenarios.

No commercially available intramedullary implants exist to determine the simulated parameters, making it impossible to validate the determined mechanical conditions experimentally. However, the general capability of fracture condition simulation has been previously demonstrated (Andres et al., 2025c; Ghiasi et al., 2017), and clinical validation through the healed fracture supports its efficacy. Furthermore, the application of a postoperative, simulated biomechanical healing control has been described in a case series and subsequently validated through animal experiments (Dailey et al., 2019; Schwarzenberg et al., 2021). Additionally, research has shown that finite element simulations of bone-implant systems can be effectively validated using experimental data, reinforcing the accuracy of these simulations in replicating physical behaviors. Our recently published paper validated our simulation through biomechanical experiments on human specimens, and the results are consistent with those from the experiments (Wickert et al., 2024).

In the aftercare of tibial plateau fractures, a primary focus with potential implications for the clinical and radiographic treatment outcomes is postoperative weight-bearing (Arnold et al., 2017; Canton et al., 2022; Thewlis et al., 2015; Williamson et al., 2018). Traditionally, long periods of non-weight bearing have been prescribed for fear of secondary fracture dislocation, intra-articular step of formation, and, ultimately, a higher risk of posttraumatic arthritis. The current surveys, as well as clinical analysis, have shown, however, that these strict non-weight-bearing regimes are increasingly being abandoned in clinical practice, and that early, permissive weight-bearing in select cases is not associated with a higher degree of complications (Arnold et al., 2017; Thewlis et al., 2015). However, a low-powered study directly measuring postoperative joint subsidence through radio stereometrics analysis has shown that higher average knee joint loading during the stance phase of gait is not associated with increased postoperative fracture fragment migration beyond 3 mm (Thewlis et al., 2015). The clinical significance of these increases in step-off formation, particularly in relation to long-term outcomes, remains unclear. The relevance of an individual simulation approach in determining this risk for secondary fracture migration and loss of reduction is evident in the second patient with medial-sided plating. This is the only patient simulated in this analysis with postoperative fracture subsidence and subsequent loosening of a screw in the plate apex, experiencing an outward pushing force running almost parallel to the fracture plane. This patient reported being permissively weight-bearing within the first weeks after the fracture and used no aids for walking, thus exposing her to at least her full body weight. Accordingly, the simulation of the case under full weight-bearing conditions showed the highest amount of fracture area, above safe ranges of interfragmentary motion according to the healing window (Lutz, 2017), confirming the simulation’s principal capability to assess situations at risk, already seen in studies (Braun et al., 2021; Braun et al., 2019). Interestingly, the fracture in this patient consolidated in this position and has led to a satisfactory result for the patient after screw removal and prescription of partial weight-bearing for 6 weeks.

The other simulated fracture situations with triple plate osteosynthesis have shown sufficient stability and safe ranges of interfragmentary motion throughout all weight-bearing levels. Accordingly, these fractures exhibited uneventful postoperative treatment courses, with no secondary loss of reduction or hardware failures. Both patients, however, required more than 8 weeks to gradually wean themselves off their walking aids. This aligns with current clinical studies and review analyses focusing on permissive weight-bearing. While it is safe to allow permissive weight-bearing, patients often require considerable time to regain full weight-bearing (Pishtiwan Hassan Shaker Kalmet et al., 2019). Simulation workflows, such as the one introduced here, can help identify patients who are safe for higher degrees of weight-bearing early during the treatment course and potentially increase their physical therapy training within their simulated safe range, guiding them toward earlier functional recovery (Braun et al., 2021; Braun et al., 2017).

The approach demonstrates the value of a simulation workflow in testing the weight-bearing associated effects on construct stability, joint reaction forces, and fracture healing. Identifying patients at risk for treatment failure early in the fracture course is a potentially significant factor in reducing the patient and socioeconomic burden of disease. On the patient’s side, early interventions, ranging from adapting the aftercare regimen to surgical treatment, are possible. Aftercare adaptations can reduce weight-bearing in situations with increased interfragmentary motion or the risk of fixation failure. In contrast, the frequency and intensity of full weight-bearing can be addressed in understimulated situations. Furthermore, individual, simulation-adjusted adaptation of ROM braces could prevent certain critical motions. If the simulation determines that healing cannot be achieved by modulation of the weight-bearing input, the stiffness of the osteosynthetic construct could be adjusted, i.e., through augmentative plating, as well as further biologic measures (autologous bone graft) in mechanically sound situations without clinical healing. By establishing this simulation as a rehabilitation and screening tool, the added socioeconomic effects can be expected through better healing rates through adapted therapy and earlier intervention in critical cases. Research on joint reaction forces in arthroplasty has shown that it is not only gait and associated weight-bearing that are putting stress on the proximal tibia, but also different movement situations that can exert high levels of force on a treated joint (Berg et al., 2014). The presented workflow and the database of reference movements can be extended to include more scenarios, such as getting out of bed, walking at different speeds, or rising from a chair, to provide a more comprehensive simulation of individual patients’ movement patterns. Furthermore, this would allow us to test the particular patient in more detail and at a higher level of injury specificity, such as high-degree flexion testing in flexion-type injuries, to adapt postoperative range of motion protocols.

Simulation approaches, such as the one introduced in this article, offer a unique opportunity to enhance our understanding of the effects of postoperative weight-bearing on osteosynthetic construct stability, fracture healing, and, ultimately, patient outcomes. Over the past 30 years, the discussion on potential weight-bearing recommendations following operative fracture treatment of the proximal tibia has remained active and controversial (Canton et al., 2022; Kubiak et al., 2013; Whitelaw et al., 1993). Determining the biomechanical effects of weight-bearing on joint mechanics without computer simulation requires both controlled weight-bearing and precise radiographic measures of joint subsidence and fracture movement early during the fracture course. Limited patient number studies combining both these techniques have been performed showing that increased amounts of weight-bearing after proximal tibia fractures can be allowed without negative radiographic and clinical consequences (Thewlis et al., 2015; The et al., 2017), but to get to a comprehensive recommendation on all types of proximal tibia fractures, while considering the many treatment and hardware placement options is not feasible with these highly complex and research-oriented techniques. Clinical studies recommending permissive weight-bearing in these patients have shown that this can be associated with a decreased short-term recovery time, but also with limitations to the amount of weight-bearing, especially at the study endpoint, when the patient converts to a total knee arthroplasty (Pishtiwan Hassan Shaker Kalmet et al., 2019). To safely test these limitations early during the fracture treatment on the individual treatment situation, but safely with simulated, different weight-bearing levels, thus not only offers the opportunity to increase our understanding of safe boundaries during fracture rehabilitation but actually can allow for individualized aftercare concerning weight-bearing with wearable techniques, in situations that would otherwise be hard to assess and control (Braun et al., 2017; Marmor et al., 2022). The clinical feasibility of our approach has been demonstrated, and it can now be further refined in larger-scale clinical studies. The model’s outcome predictions enable the development of tailored post-operative care plans, ensuring high-risk patients receive the necessary attention to prevent complications. Hospitals can allocate resources more efficiently, reducing healthcare costs by preventing complications and readmissions. Predictive insights enable clinicians to implement early intervention strategies, thereby improving patient outcomes. Additionally, the findings can inform enhanced recovery after surgery protocols, optimizing recovery times and reducing hospital stays.

## 5 Limitations

While the study provides insightful results, it also offers opportunities for further research and refinement. Including a relatively small number of patients provides a focused initial investigation, paving the way for future studies with larger cohorts to enhance the robustness and applicability of the results. While the simulations do not capture the full biological context of the injury, including concomitant soft tissue damage and the effects of the ligamentous injury on joint stability, they open the door to more comprehensive modeling that incorporates these factors. In addition, the study highlights an important area for further investigation: determining the precise level of mechanical stimuli within the fracture zone that is optimal for healing.

The inclusion of a relatively small number of patients enables an initial analysis and serves as preparation for future studies with larger cohorts in order to improve the validity and applicability of the results. In addition, no formal power analysis was performed, as the study was designed as a feasibility and proof-of-concept investigation. The cohort of the uninjured reference group consisted of young individuals without comorbidities, which does not correspond to the typical patient group with tibial plateau fractures.

Although the simulation predicted biomechanical overload in Case #2 consistent with screw loosening, delayed healing in this patient was likely multifactorial. Age, comorbidities, balance deficits, and construct choice all contributed, and the model cannot fully isolate mechanical loading as the sole determinant. Moreover, clinical validation was limited to retrospective case comparison, which may invite confirmation bias; prospective validation in larger patient populations is required.

No sensitivity analysis was performed beyond this. While the simulations do not capture the full biological context of the injury—including concomitant soft tissue damage, vascularity, or the effects of ligamentous injury on joint stability—they open the door to more comprehensive modeling that incorporates these factors. This is a feasibility study to demonstrate the technical plausibility and clinical relevance of combining motion capture, musculoskeletal simulation, and finite element modeling.

## 6 Conclusion

This study introduces a patient-specific simulation framework for evaluating postoperative weight-bearing after tibial plateau fractures. By integrating patient imaging with reference motion data, the approach enables safe, non-invasive testing of individual loading scenarios and has the potential to inform personalized rehabilitation protocols. Beyond walking, the model can be extended to incorporate other everyday movements such as stair climbing or rising from a chair, broadening its clinical utility. Future research with larger cohorts will be essential to refine predictive accuracy and establish its value as a clinical decision-support tool. Ultimately, the goal is to define safe postoperative boundary conditions for loading and range of motion, supporting fracture healing while preserving implant integrity.

## Data Availability

The raw data supporting the conclusions of this article will be made available by the authors, without undue reservation.
