# Supplementary material for: Predicting the effect of individual weight-bearing on tibial load and fracture healing after tibial plateau fractures–introduction of a biomechanical simulation model
Source: Front Bioeng Biotechnol. 2025 Sep 25;13:1659029. doi: 10.3389/fbioe.2025.1659029 (PMC12507901; doi:10.3389/fbioe.2025.1659029)
Supplement: Supplementary file 1 [file DataSheet1.docx]

**Supplementary Material**

**A:**

This part provides a step-by-step overview of the workflow used to process motion and ground reaction force data and perform musculoskeletal simulations.

**A1. Data Acquisition and Synchronization**

- Motion Capture: Full-body kinematics were recorded at 240 Hz using the Xsens™ Link system (Figure S1), which features 17 inertial measurement units (IMUs) placed according to the manufacturer’s guidelines.
- Sensors placement: bilateral thighs, shanks, feet, shoulders, hands, and lower and upper arms. Additionally, on the pelvis, trunk, and head.
- These miniature inertial measurement units contain
  - - a 3D magnetometer to measure the 3D geomagnetic field,
    - a 3D velocity gyroscope to measure the 3D angular velocity,
    - a linear accelerometer to measure the 3D acceleration and
    -
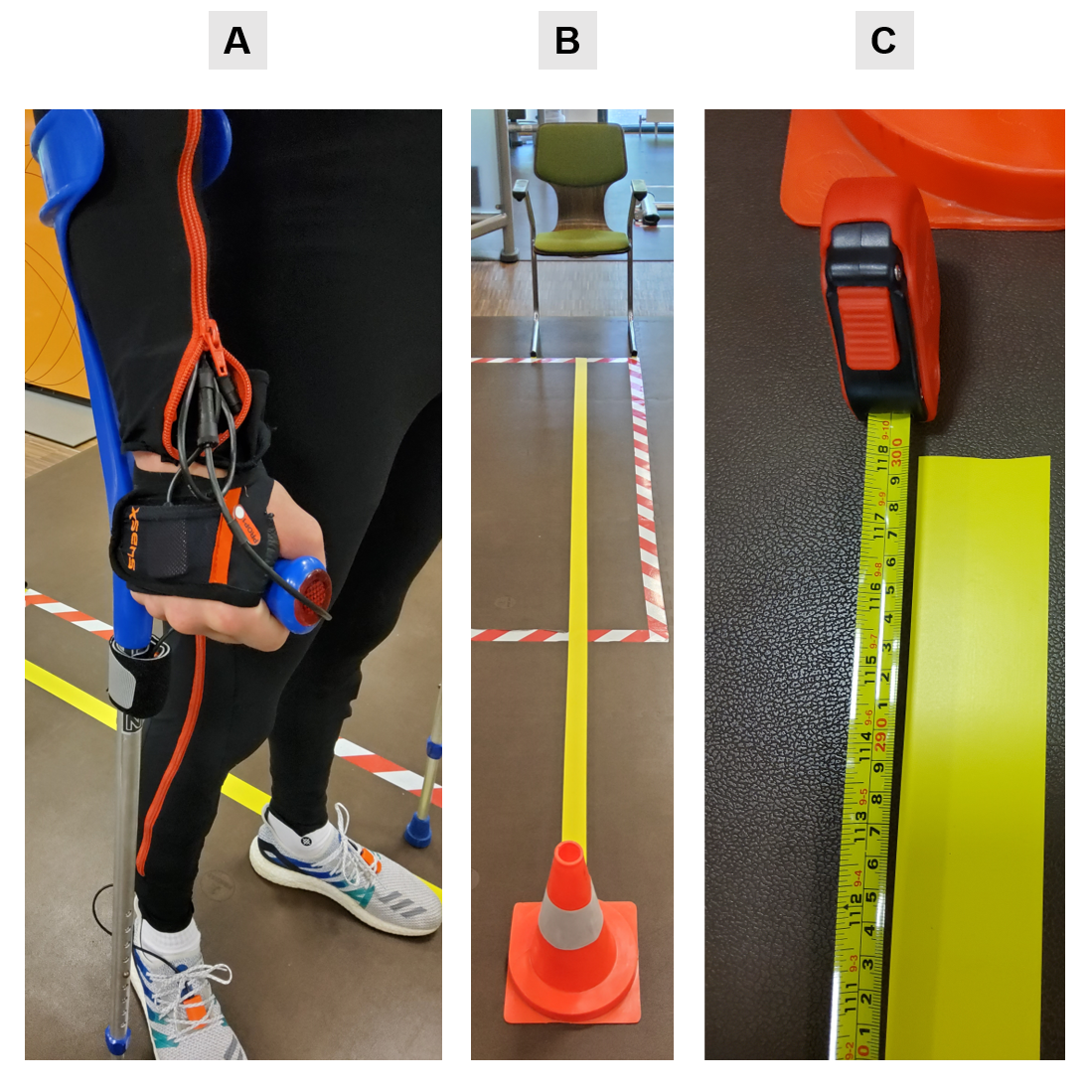
a barometer, to measure the atmospheric pressure

Figure S1: Xsens^TM^ Link system (A), setup for the Timed Up and Go Test (C), and exact measurement of a 3-meter walking distance.

- Calibration of Xsens**™**: utilizing Magnetic Field Mapping (MFM) and static upright pose calibration to ensure accurate segment orientation and drift correction**.** Xsens™ system utilizes 6-DOF (Degrees of Freedom) models**.**
- Ground Reaction Forces (GRFs):
  - Moticon™ insoles (Figure S2) recorded GRFs and plantar pressure distribution at a sampling rate of 100 Hz. The insoles consist of 16 pressure sensors, three acceleration sensors, three angular sensors, and one temperature sensor.
- Novel™ loadpads recorded force distribution at 100 Hz and were used to validate Moticon™ measurements during both static calibration and dynamic gait trials.
- Synchronization: All devices were synchronized using a hardware trigger. Post-processing synchronization was verified using gait event detection (heel strike, toe-off) and cross-correlation of force curves across systems.


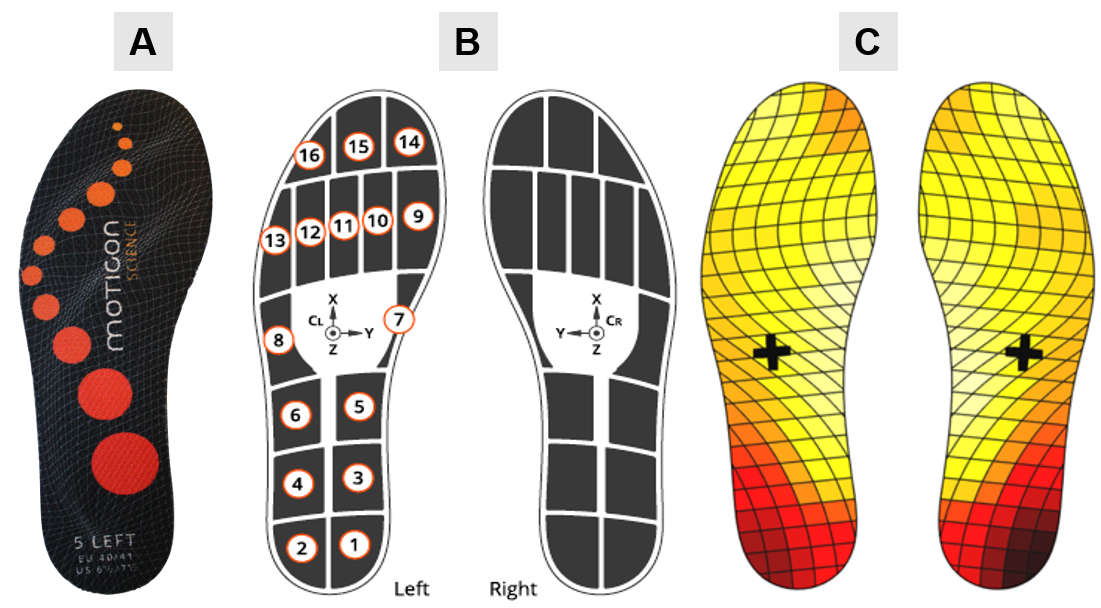
Figure S2: Moticon^TM^ sensor insole (A), Distribution of pressure sensors and position of the coordinate system (B), Live display of pressure distribution while standing (C).

**A2. Data Processing**

- Xsens™ data were exported as .bvh (BioVision Hierarchy) files and imported into AnyBody™ Modeling System using the AMMR (AnyBody™ Managed Model Repository). An explicit explanation video for the Xsens-AnyBody Workflow: https://base.movella.com/s/article/AnyBody-Xsens-workflow-webinar-kinetic-modeling-on-the-field?language=en_US
- The kinematic data were used to drive the musculoskeletal model, with individual models scaled based on each participant’s anthropometric data.
- GRF data from Moticon™ insoles, validated by Novel™ loadpads (see Figure S3), were imported as .txt files containing timestamps, Ground reaction force (GRF), and center of pressure (CoP).
  - These data were imported into AnyBody™ using:
    - AnyInputFile to load the text file
    - AnyFunInterpol to create a time-dependent interpolation function for each GRF and CoP data stream
    - The GRFs were applied to the foot segment in the model via a time-dependent force vector.
    -
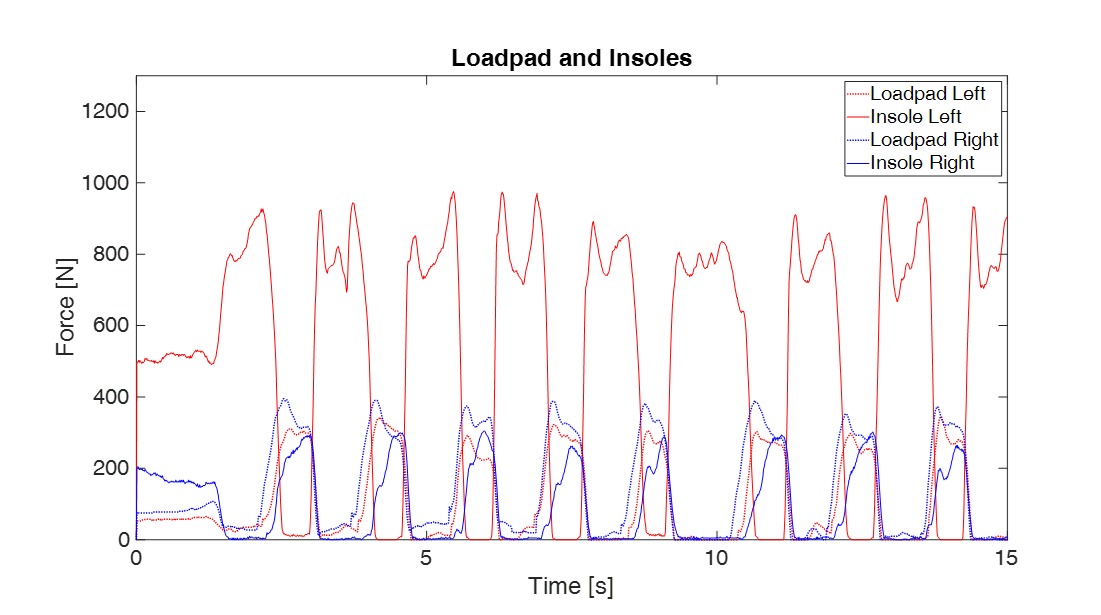
The center of pressure coordinates were used to define the dynamic point of force application, ensuring the force was applied at the correct anatomical location during each time step.

Figure S3: Simultaneous measurement of the sensor insoles with the loadpads during a partial weight-bearing measurement of 20 kg on the right leg.

**A4. Validation and Quality Control**

- Force measurements from the Novel™ loadpads were used to confirm the accuracy of load distribution and timing captured by the Moticon™ insoles.
- Simulated joint force outputs were compared with known values from the OrthoLoad™ database to ensure plausibility, although no direct validation was performed in this study.
- Only trials that showed consistent load adherence and minimal signal noise were included in the final analysis.
- The systems used for inertial motion capture (Xsens™), insole-based force measurement (Moticon™), and musculoskeletal modeling (AnyBody™) provide a validated and reproducible workflow, as demonstrated in previous studies (Burns et al., 2019; Andersen et al., 2009; Muro-de-la-Herran et al., 2014).


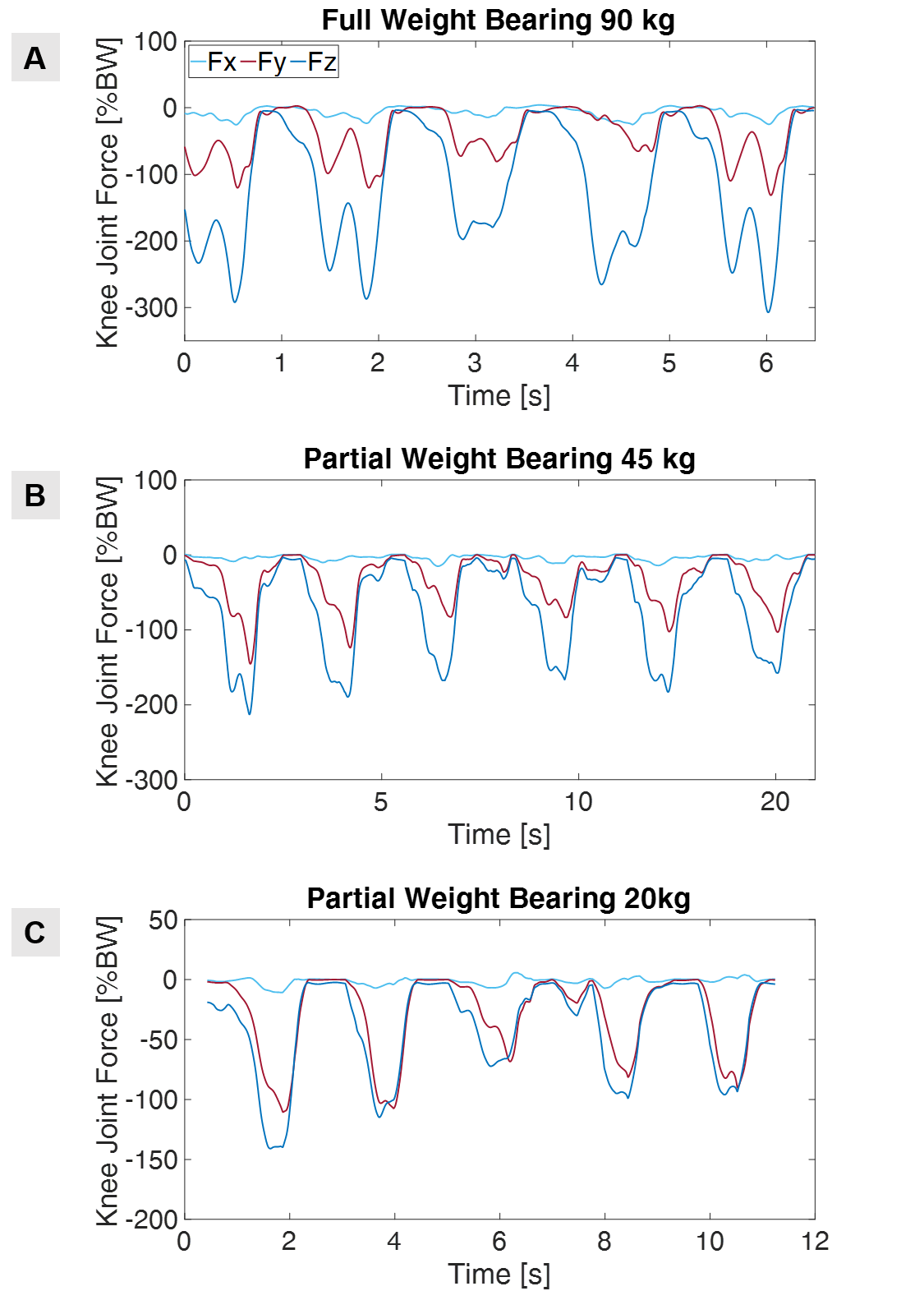
Figure S4: Knee joint force during walking of a healthy subject for (A) full body weight bearing, (B) partial weight bearing of 50 % body weight, and (C) 20 kg partial weight bearing.

**B:**

This part outlines the construction and execution of the patient-specific finite element (FE) models used to assess implant stress and interfragmentary strain under varying loading conditions.

**B1. Software and Workflow Overview**

- All FE models were developed using ScanIP™ (Synopsys) for segmentation and meshing, and simulated in ABAQUS/Standard™ (Dassault Systèmes).
- Patient-specific models were based on postoperative CT scans, imported in DICOM format. The scans were performed using a Siemens SOMATOM Definition Edge CT scanner with the following parameters:
- Body part examined: Extremity
- Slice thickness: 0.75 mm
- Spacing: 0.3125 x 0.3125 x 0.5 mm
- Reconstruction diameter: 160 mm
- Convolution kernel: B26s
- Energy settings (kVp): 120 kVp
- X-ray tube current: 72 mA
- Exposure time: 1000 ms
- Distance source to detector: 1085.6 mm
- Distance source to patient: 595 mm
- Rotation direction: Clockwise (CW)
- Patient position: Feet first supine (FFS)
- Image resolution: 512 x 512 x 541 voxels
- Pixel spacing: 0.3125\0.3125
- The workflow included segmentation, 3D reconstruction, mesh generation, assignment of material properties, and application of boundary conditions and external loads.

**B2. Geometry and Meshing**

- Segmentation was performed using semi-automatic thresholding and manual refinement in ScanIP™.
- The tibia, implant (plate and screws), and fracture gap were modeled as separate parts.
- **Mesh type**: 10-node quadratic tetrahedral elements (C3D10).
- **Mesh density**: Refined in areas near the fracture site.
  - Element volume - Mean: 0.1646 mm³
  - Element volume - Min: 2.756e-04 mm³
  - Element volume - Max: 1.392 mm³
- **Mesh quality assessment**: Quality inspection, convergence tested by comparing results across three mesh densities (coarse, medium, fine).

**B3. Material Properties**

- **Bone**: Material properties are assigned based on postoperative CT scans using a density-modulus mapping approach, informed by the results of two review papers (Helgason et al., 2007; Knowles et al., 2016).
  - Workflow:
    - Raw CT data is mapped to bone mineral density (BMD) using a six-rod calibration phantom. Histograms of Hounsfield units (HU) values are generated for each rod, and the peaks of these histograms are used as calibration points to define a linear relationship between HU and BMD.
    - The BMD values are converted to ash density using literature-based linear relationships for hydroxyapatite phantoms
    - Ash density is converted to apparent density
    - Apparent density is used to derive material parameters using tibia-specific relationships.
  - For the tibia, we use the values from Rho et al. (Rho et al., 1995) and Edwards et al. (Edwards et al., 2013).
  - Poisson’s ratio set to 0.3.
- **Implant (Ti-6Al-4V)**: E = 110 GPa, ν = 0.3 (Imam et al., 1996)
- **Fracture gap (soft callus)**: E = 0.3 MPa, ν = 0.3 (Claes et al., 1999)

**B4. Boundary Conditions and Load Application**

- **Distal node set**: Fixed in all translational degrees of freedom.
- **Proximal node set**: Knee joint forces from AnyBody™ simulations were applied as time-independent vectors for each loading condition (20 kg, 50% body weight, full weight-bearing), scaled to the body weight (Figure S4).
- Forces applied at the tibial plateau, considering magnitude and direction relative to anatomical axes.

**B5. Solver Settings**

- Simulations performed using ABAQUS/Standard™ (implicit) solver.
- Nonlinear static analysis with automatic time incrementation.
- **Convergence tolerance**: Force residuals < 1% of applied load.
- Newton–Raphson method with line search used for nonlinear solution.
